# Human umbilical cord blood mononuclear cells transplantation for perinatal brain injury

**DOI:** 10.1186/s13287-022-03153-y

**Published:** 2022-09-05

**Authors:** Yufeng Xi, Guang Yue, Shuqiang Gao, Rong Ju, Yujia Wang

**Affiliations:** 1 Department of Neonatology, Chengdu Women’s and Children’s Central Hospital, School of Medicine, University of Electronic Science and Technology of China, Chengdu, 611731 China; 2 Department of Dermatology, State Key Laboratory of Biotherapy, West China Hospital, Sichuan University, Chengdu, 610041 China

**Keywords:** Human umbilical cord blood mononuclear cells, Perinatal brain injury, Transplantation

## Abstract

Perinatal brain injury is a leading cause of death and disability in children. Hypoxic-ischemic encephalopathy in full term infants, and white matter injury in premature infants are most known brain injury in perinatal period. Human umbilical cord blood mononuclear cells contain hematopoietic stem cells, mesenchymal stem cells, endothelial progenitor cells, lymphocytes, monocytes, and so on. Human umbilical cord blood mononuclear cells have many biological functions, such as nerve and vascular regeneration, anti-apoptosis, anti-inflammation, and immune regulation. Human umbilical cord blood mononuclear cells transplantation has achieved significant efficacy and safety in animal and clinical trials for the treatment of perinatal brain injury. We will review human umbilical cord blood mononuclear cells transplantation for perinatal brain injury in this review.

## Background

Perinatal brain injury is a main cause of death and disability in children. The pathogenesis of perinatal brain injury is considerable complex, involving hypoxia–ischemia, inflammation, infection, reactive oxygen species, excitotoxic injury, and so on [1–3]. Perinatal brain injury may result in significant long-term neurodevelopmental impairment, such as cognitive, neurologic, motor, and sensory disability [4]. At present, mild hypothermia therapy is the most effective way to attenuate brain injury in the newborn [5]. Unfortunately, this treatment is only partially neuroprotective and can only be used to treat hypoxic-ischemic encephalopathy in full term infants [5]. Therefore, there is an urgent need for effective and safe therapeutic strategies.

Stem cells have been extensively researched to treat neurological conditions as they have regenerative, anti‐inflammatory, and neuroprotective properties [6]. Umbilical cord blood (UCB) is an important source of stem cells. Human UCB-derived stem cells have generated significant attention in regenerative medicine [7]. Human UCB not only contains a variety of stem cells, but also contains lymphocytes and monocytes, and they are all called mononuclear cells. In this review, we will discuss the therapeutic effect of umbilical cord blood mononuclear cells in perinatal brain injury, including hypoxic-ischemic encephalopathy (HIE) and white matter injury (WMI).

## Main cell types in the umbilical cord blood

UCB refers to the blood in the umbilical cord and the blood vessels near the fetal side of the placenta at birth. Human UCB mononuclear cells (hUCB-MNCs) contain hematopoietic stem cells (HSCs), mesenchymal stem cells (MSCs), endothelial progenitor cells (EPCs), lymphocytes, monocytes, and so on [8].

### HSCs

HSCs [9] are one of the most abundant stem cells in UCB. UCB-derived HSCs have a less mature phenotype than adult bone marrow and peripheral blood, but present a strong ability to proliferate in response to cytokines in vitro [10]. CD34 is the most widely used surface marker of HSCs [11]. HSCs are specialized stem cells, which can differentiate into cells of the blood system and immune system [9]. Specifically, HSCs can differentiate into various blood cells in myeloid system (monocytes and macrophages, neutrophils, basophils, eosinophils, erythrocytes, megakaryocytes/ platelets, dendritic cells) and lymphatic system (T, B and natural killer cells) [12]. So far, they are mainly used to treat blood system diseases, such as leukemia, aplastic anemia, and thalassemia. In a mouse model of neonatal stroke, the cerebral blood flow and blood vessel diameter in the area of cerebral infarction transiently increased after CD34 ^+^HSCs were given, due to the release of growth factors to stimulate neurogenesis and angiogenesis [13].

## MSCs

MSCs are stromal cells that have the capacity to self-renew and present multilineage differentiation [14]. MSCs can be isolated from a variety of tissues, such as umbilical cord, bone marrow, and adipose tissue [14]. They can not only differentiate into a variety of mesoderm derived cells, such as adipocytes, osteoblasts, and muscle cells, but also differentiate into endoderm derived hepatocytes and ectoderm derived nerve cells. HUCB-derived MSCs have been widely used in the clinical research of central nervous system diseases such as cerebrovascular diseases, neurodegenerative diseases, spinal cord injury, and neonatal HIE [15–18]. In recent years, however, more and more evidence suggests that MSCs exert their therapeutic effects in brain injury via paracrine mechanisms [19], including the release of extracellular vesicles, such as microvesicles, exosomes [20–22]. Drommelschmidt et al. found that MSC-derived extracellular vesicles ameliorated inflammation-induced preterm brain injury via preventing neuronal cell death, restoring of white matter microstructure, reducing of gliosis and long-term functional improvement [23]. On the one hand, umbilical cord blood mesenchymal stem cells promote the proliferation of hematopoietic stem cells by secreting a variety of cytokines with hematopoietic support [24, 25]; on the other hand, umbilical cord blood mesenchymal stem cells express adhesion molecules that interact with hematopoietic cells, regulate the adhesion between hematopoietic cells and stromal cells, and play an important role in hematopoietic cell implantation and homing [24]. Many experiments have shown that the combined transplantation of UCB-derived MSCs and HSCs can improve the success rate of hematopoietic stem cell transplantation [26–28]. Intracerebral transplantation of mesenchymal stem cells derived from human umbilical cord blood alleviated hypoxic-ischemic brain damage (HIBD) in rat neonates via migrating to damaged brain tissues to inhibit neuronal apoptosis [29].

### EPCs

EPCs are the precursor cells of vascular endothelial cells [30]. They are a group of immature endothelial cells with migratory characteristics and can further proliferate and differentiate [31]. They lack the characteristic phenotype of mature endothelial cells and can’t form lumen like structure. Their function is mainly to participate in the angiogenesis of postnatal ischemic tissue and the repair after vascular injury [31]. There are both hematopoietic stem cell surface markers, such as CD34, CD133, and endothelial cell surface marker VEGFR-2 on the surface of EPC cells [32]. Studies have shown that endothelial progenitor cells play an important role in cardiovascular and cerebrovascular diseases, ischemic diseases [31, 33]. In a rat model of HIBD, the researchers suggested that EPCs administration suppressed cellular apoptosis, neuroinflammation, and astrocytic reaction, restored cerebral capillary density, and improved neuronal cell survival [34]. In addition, CD133^+^EPCs have therapeutic potential in neonatal hypoxic-ischemic encephalopathy via migrating to damaged brain tissues, resulting in recovered motor function and decreased lesion size [35].

### Lymphocytes

Lymphocytes are the core of immune response. According to the origin, morphological structure, surface markers and immune function of lymphocytes, they can be divided into T cells, B cells and NK cells [36]. Compared with adult peripheral blood, UCB contains a similar percentage of B lymphocytes and a reduced percentage of T lymphocytes [36]. In addition, UCB-derived lymphocytes are phenotypically and functionally immature [37]. In a rat model of neonatal HIBD, hUCB-derived T regulatory cells (Tregs) were able to reduce motor deficits, CD4^+^ T cell infiltration into the brain, and reduce microglial activation [38]^.^

### Monocytes

Monocytes are immature phagocytes circulating in the blood [39]. As an antigen presenting immune cell [40], they can phagocytize and degrade microorganisms and particulate matter. Monocytes can differentiate into several different cell types. Monocytes can differentiate into dendritic cells, macrophages, and osteoblasts [40, 41]. In mouse brain organotypic slice of oxygen and glucose deprivation, cord blood derived CD14^+^ monocytes protected neuronal cells from death and reduced glial activation [42].

## Neonatal HIE

HIE is an important cause of permanent damage to central nervous system that may result in neonatal death or manifest later as mental retardation, epilepsy, cerebral palsy, or developmental delay [43]. The primary cause of this condition is systemic hypoxemia and/or reduced cerebral blood flow with long-lasting neurological disabilities and neurodevelopmental impairment in neonates.

### Possible mechanisms of action of UCB-MNCs in HIE

#### Anti-apoptosis

One of the main mechanisms of neonatal hypoxic-ischemic brain damage is apoptosis. The anti-apoptosis effect of human UCB-MNCs was observed in numerous studies. Yu et al. [44] demonstrated that hUCB-derived CD34^+^ cells transplantation can ameliorate the neural functional defect and apoptosis by inhibiting the expression of apoptosis-related genes: TNF-α, TNFR1, TNFR2, CD40, Fas, and reducing the activation of nuclear factor kappa B (NF-κB) in damaged brain. Hattori et al. [45] also confirmed that a single intraperitoneal injection of UCB-MNCs 6 h after a cerebral ischemic injury was associated with a reduction in numbers of apoptosis and oxidative stress marker-positive cells. The number of cells positive for the apoptosis markers active caspase-3 and apoptosis-inducing factor decreased by 53 and 58%, respectively. In addition, UCB-MNCs can secrete neurotrophic factors by paracrine to promote nerve cell growth and reduce apoptosis. Yasuhara et al. [46] revealed that the level of neurotrophic factors, such as GDNF, NGF and BDNF were increasing in brain tissue of those hypoxic-ischemic injury rats that received UCB-MNCs, and the expression of apoptosis marker caspase-3 decreased. The above results have shown that UCB-MNCs could downregulate the expression of apoptosis-related genes or upregulate neurotrophic factors to inhibit apoptosis.

### Nerve regeneration

Promoting nerve regeneration in the damaged area is a way to treat HIE. Many studies have shown that transplanted UCB-MNCs proliferate and differentiate into neurons and glial cells to replace damaged cells. Some studies suggested that homing of UCB-MNCs to the lesion site can improve brain damage. A study [47] shown that transplantation of human umbilical cord blood-derived mononuclear cells in a rat model of perinatal brain damage leads to both incorporation of these cells in the lesioned brain area and to an alleviation of the neurologic effects. In mechanism, Rosenkranz et al. [48] demonstrated that SDF-1 /CXCL12 mediated the homing of UCB-MNCs to the lesion site. Yu et al. [44] found that transplantation of human umbilical cord blood CD34+ cells can promote nerve and vascular regeneration in rat brain after HI injury. In addition, Wang et al. [49] indicated that UCB-MNCs may promote neural stem cell (NSC) proliferation and alleviate brain injury in HI neonatal rats via Shh signaling. In summary, UCB-MNCs translation can migrate to the damaged area to promote nerve regeneration.

### Neurovascular regeneration

Promoting angiogenesis in hypoxic-ischemic brain injury area is a new research direction in the treatment of HIE. Cho et al. [50] found that UCB-MNCs transplantation can activate IL-8-mediated angiogenesis signal pathway, promoting angiogenesis in the injured area and reducing the area of cerebral infarction. Besides, Yu et al. [44] also confirmed UCB-MNCs transplantation can promote vascular regeneration. Above of all, UCB-MNCs transplantation can active angiogenic related pathway to promote neurovascular regeneration.

### Anti-inflammatory and immunity regulation

When hypoxic-ischemic injury occurs, microglia in the brain are activated. The activated microglia migrate to the injury site and release proinflammatory factors together with astrocytes, resulting in inflammatory response. Many studies have shown that UCB-MNCs transplantation reduce the activation of microglia and astrocytes. The researchers co-cultured UCB-MNCs with oxygen–glucose deprivation (OGD) mouse brain slice, then they found that the activation of microglia and astrocytes decreased, and the expression of anti-inflammatory factor IL-10 increased. Zhang et al. [16] indicated that transplantation of CB-MNCs after HI reduced astrogliosis and increased the proportion of mature oligodendrocytes, prevented neuronal loss in the striatum. In summary, inhibiting of microglia and astrocytes activation can downregulate inflammatory response. McDonald et al. [38] demonstrated that UCB and specific cell types found in UCB, namely EPCs, Tregs, and monocytes, can modulate the peripheral and central immune response. The ability of different UCB cells to modulate the central T cell responses and reduce microglial activation is varied. These data suggest that UCB cells may act directly to modify the entry of immune cells into the brain. In addition, EPCs also significantly reduced cortical cell death, returned CD4^+^ T cell infiltration to sham levels, and reduced the peripheral Th1-mediated pro-inflammatory shift. In a word, UCB-MNCs can improve brain injury through immune regulation and downregulating inflammatory response.

## UCB-MNCs transplantation in animal models of HIE

Many animal experiments have shown that CB-MNCs transplantation can alleviate brain injury. Yu et al. [44] confirmed that transplantation of human umbilical cord blood CD34+ cells, including hematopoietic stem cell/endothelial progenitor cells, can ameliorate the neural functional defect and reduce apoptosis and promote nerve and vascular regeneration in rat brain after hypoxic-ischemic injury. The effects of transplantation of CD34+ cells were comparable to that of MNCs in neonatal hypoxic-ischemia rat model. Rosenkranz et al. [48] found that SDF-1/CXCR4 axis is of major importance for cell homing. Transplanted UCB-MNCs expressing the SDF-1 receptor CXCR4 migrated to the lesion site within one day. Inhibition of SDF-1 by application of neutralizing antibodies in vivo resulted in a significantly reduced number of UCB-MNCs at the lesioned area. Wang et al. [49]. found that the hypoxic-ischemic + UCB-MNCs group had significantly less neuronal loss in the cortex and CA1 sector of the hippocampus compared to the hypoxic-ischemic + PBS group. Further results [49] indicate that UC-MSC may promote NSC proliferation and alleviate brain injury in neonatal HIBD rats via Shh signaling. Aridas et al. [51] reported that autologous UCB mononuclear cell treatment restored normal brain metabolism following perinatal asphyxia, and reduced brain inflammation, astrogliosis and neuronal apoptosis in the newborn lamb, supporting its use as a neuroprotective therapy following asphyxia.

Many studies have also shown that cell transplantation can improve the motor ability and memory of animal. Zhang et al. [16] show that transplantation of UC-MSCs or CB-MNCs after hypoxic-ischemic reduced astrogliosis, prevented neuronal loss in the striatum, and markedly improved functional brain outcomes after a 28-day recovery period. Moreover, treatment with UCB-MNCs increased the proportion of mature oligodendrocytes and improved myelination in cortical areas. Cho et al. [50] confirmed that the administration of UCB-MNCs after hypoxic-ischemic upregulates angiogenic gene expression and resultant angiogenesis via the CXCL2-mediated activation of the p38 pathway, resulting the decrease of cerebral infarction area and the improvement of motor. Meier et al. [47] suggested that after transplantation of human umbilical cord blood-derived mononuclear cells, spastic paresis was largely alleviated, resulting in a normal walking behavior. Pimentel-Coelho et al. [52] demonstrated that intraperitoneal transplantation of UCB-MNCs, 3 h after the hypoxic-ischemic insult, resulted in better performance in two developmental sensorimotor reflexes, in the first week after the injury. They also showed a neuroprotective effect in the striatum, and a decrease in the number of activated microglial cells in the cerebral cortex of treated animals. Yasuhara et al. [46] examined whether the same blood–brain barrier manipulation approach increases the therapeutic effects of intravenously delivered UCB-MNCs in a neonatal HIBD model. They compared vehicle alone, mannitol alone, UCB-MNCs alone or a combination of mannitol and UCB-MNCs, then they revealed elevated levels of GDNF, NGF and BDNF in those that received hUCB cells alone or when combined with mannitol compared with those that received vehicle or mannitol alone, with less impaired in motor asymmetry and motor coordination. Greggio et al. [53] found that intra-arterially transplanted hUCB mononuclear cells significantly improved learning and long-term spatial memory impairments when evaluated by the Morris water maze paradigm at 9 weeks post-hypoxic-ischemic.

However, some studies also showed that cells did not improve hypoxic-ischemic brain damage. Hattori et al. [45] suggested that transplantation of UCB-MNCs did not induce long-term morphological or functional protection.

In summary, transplantation of UCB-MNCs can not also promote the repair of HIBD, but also improve the motor ability and spatial memory of animal. The role and mechanism of hUCB-MNCs transplantation in the treatment for HIE are shown in Table 1.

**Table 1:** Human umbilical cord blood mononuclear cells transplantation for perinatal brain injury

Monocyte type	Timing of treatment	Cell number	Mode of administration	Effect	Mechanism	References
CD34 + cells	7 days after HI	1.5*10^4^	Tail vein	Ameliorated the neural functional defect and reduced apoptosis and promoted nerve and vascular regeneration	inhibited the expression of apoptosis-related genes, such as TNF-α, TNFR1, TNFR2, CD40, Fas	44
UCB-MNCs	6 h after HI	1*10^7^	Intraperitoneal	Didn’t induce long-term morphological or functional protection	inhibited apoptosis and oxidative stress	^45^
UCB-MNCs	7 days after HI	1.5*10^3^	Jugular vein	Less impaired in motor asymmetry and motor coordination	Up-regulation of neurotropic factor, such as GDNF, NGF and BDNF	^46^
UCB-MNCs	24 h after HI	1*10^7^	Intraperitoneal	Improved spastic paresis	Promoting homing of MNCs to the lesion site	^47^
UCB-MNCs	24 h after HI	1*10^7^	Intraperitoneal	Ameliorated brain damage	SDF-1 /CXCL12 mediated the homing of MNCs to the lesion site	^48^
UCB-MNCs	24 h after HI	3*10^6^	Paracele	Promoted NSC proliferation and alleviated brain injury	Actived hedgehog signaling mediated cell proliferation pathway	^49^
UCB-MNCs	7 days after HI	3*10^7^/kg	Intraperitoneal	Promoted angiogenesis in the brain injury area and reduced the area of cerebral infarction	Actived the IL-8-mediated angiogenic pathway	^50^
UCB-MNCs	6 h after HI	1*10^7^	Paracele	Improved neonatal rat memory	Reduced astrogliosis, prevented neuronal loss in the striatum	^16^
UCB-MNCs	12 h after perinatal asphyxia	1*10^8^	Brachial artery catheter	Reduced clinical markers of brain damage following perinatal asphyxia	Reduce neuroinflammation and neuronal apoptosis	
UCB-MNCs	3 h after HI	2*10^6^	Intraperitoneal	Ameliorated brain damage	Reduced activated caspase-3-mediated cell death in the striatum, and microglial activation in the cerebral cortex	^52^
UCB-MNCs	24 h after HI	1*10^7^	Intra-arterial	prevented long-term cognitive deficits	unknown	^53^

## WMI in premature infants

WMI is the most frequent form of preterm brain injury [54]. The most serious outcome is paraventricular leukomalacia in preterm infants, which will cause neurological sequelae in children, such as cerebral palsy, abnormal audio-visual function, and cognitive impairment.

In an earlier study [55], researchers induced neonatal brain lesions using intracranial injections of ibotenate, then UCB-MNCs were injected either intraperitoneally (i.p.) or intravenously (i.v.) soon or 24 h after ibotenate injection. Disappointingly, the intraperitoneal injection of high amounts of UCB-MNCs aggravated WMI and was associated with systemic inflammation. However, other researchers suggested UCB-MNCs can effectively ameliorate white matter injury induced by LPS. Paton et al. [56] used LPS intrauterine induction to establish preterm sheep WMI model, then every animal received 10^8^ UCB-MNCs intravenously to treat. They found that UCB-MNCs therapy attenuated cell death and inflammation, and recovered total and mature oligodendrocytes. In another study conducted by Paton et al. [57], they compared the neuroprotective benefits of UCB-MNCs versus MSCs in a large animal model of inflammation-induced preterm brain injury. This study suggested that UCB-MNCs significantly decreased cerebral apoptosis and protected mature myelinating oligodendrocytes, but MSCs did not. Li et al. [58] reported that UCB cells administration at 12 h after HI reduces preterm white matter injury, via anti-inflammatory and antioxidant actions.

In summary, at present, there are few studies on UCB-MNCs transplantation in the treatment of brain white matter injury, but it is still a promising treatment.

## Clinical application in perinatal brain injury

Several clinical studies on the systemic administration of UCB cells (UCBCs) for neonatal HIE have been published thus far. Almost all studies demonstrated the beneficial effects of UCBCs treatment. Tsuji et al. [59] conducted a single-arm clinical study to examine the feasibility and safety of intravenous infusion of autologous UCBCs for newborns with HIE. When a neonate was born with severe asphyxia, the UCB was collected, volume-reduced, and divided into three doses. The processed UCB was infused at 12–24, 36–48, and 60–72 h after the birth. The designed enrolment was six newborns. All six newborns received UCBC therapy strictly adhering to the study protocol together with therapeutic hypothermia. At 30 days of age, the six infants survived without circulatory or respiratory support. At 18 months of age, neurofunctional development was normal without any impairment in four infants and delayed with cerebral palsy in two infants.

Cotten CM et al. compared cell recipients’ hospital outcomes (mortality, oral feeds at discharge) and one year survival with Bayley III scores ≥ 85 in 3 domains (cognitive, language, and motor development) with cooled infants who did not have available cells. Cell recipients and concurrent cooled infants had similar hospital outcomes. Thirteen of 18 (74%) cell recipients and 19 of 46 (41%) concurrent cooled infants with known 1-year outcomes survived with scores ≥ 85. Collection, preparation, and infusion of fresh autologous UCB cells for use in infants with HIE is feasible. A study [60] under consideration (ACTRN12619001637134) aims to evaluate the safety and feasibility of autologous umbilical cord blood collection and mononuclear cell infusion in extreme premature infants. The primary aims of this study are to test (a) feasibility of autologous cord blood collection and cell retrieval following processing from extremely premature infants; and (b) safety of autologous UCBCs administration in eligible extremely preterm infants. It is found that there are 18 clinical studies in progress which are registered in www.clinicaltrials.gov.

## Conclusion

HUCB-MNCs contain HSCs, MSCs, EPCs, lymphocytes, monocytes, and so on. Unlike cell therapy using a single cell type, UCB-MNCs therapy involves multiple cell types with various functions, including anti-inflammatory, immunomodulatory, nerve regeneration, neurovascular regeneration, and anti-apoptosis. However, it is not clear that how different cell type cooperate with each other to act treating effect. Co-culture with MSCs is helpful for HSCs expansion in vitro*,* while co-transplantation with MSCs contributes to HSCs implantation and homing [11, 27, 61]. Immune regulatory cells in umbilical cord blood may facilitate HSCs transplantation tolerance. Under certain circumstances, MSCs can regulate the differentiation of monocytes, maturation of dendritic cells, and lymphocyte proliferation [62–64]. In addition, MSC derived exosomes could enhance and stabilize of UCB-derived Tregs suppressive function [65]. Transplantation of UCB-MNCs can promote the repair of HIBD and improve the motor ability and spatial memory in neonatal HIBD rats. In addition, transplantation of UCB-MNCs can ameliorate white matter injury. Some clinical trials have shown that UCB-MNCs transplantation achieved significant efficacy and safety for HIE treatment. Above all, UCB-MNCs transplantation have a good application prospect in the treatment of perinatal brain injury.

## Data Availability

Data and materials were available in the manuscript.

## Abbreviations

UCB: Umbilical cord blood
hUCB-MNCs: Human umbilical cord blood mononuclear cells
HSCs: Hematopoietic stem cells
MSCs: Mesenchymal stem cells
EPCs: Endothelial progenitor cells
HIE: Hypoxic-ischemic encephalopathy
HIBD: Hypoxic-ischemic brain damage
OGD: Oxygen-glucose deprivation
NF-κB: Nuclear factor kappa B
NSC: Neural stem cell
WMI: White matter injury
